# Influence of The Cusp-Overlap and three cusps coplanar techniques on new-onset conduction disturbances following transcatheter aortic valve implantation

**DOI:** 10.1007/s12928-026-01276-0

**Published:** 2026-04-04

**Authors:** Léo Lemarchand, Raphael Grolleau, Dominique Boulmier, Guillaume Leurent, Jacques Tomasi, Abdelkader Bakhti, Sam Sharobeem, Maxime Nolf, Gwenaelle Sost, Marielle Le Guellec, Hervé Le Breton, Vincent Auffret

**Affiliations:** 1 CHU Rennes Service de Cardiologie, Université de Rennes 1, Inserm LTSI U1099, Rennes, F 35000 France; 2 CHU Rennes Service de Chirurgie thoracique et cardio-vasculaire, Université de Rennes 1, Inserm LTSI U1099, Rennes, F 35000 France; 3 Service de gériatrie, CHU Rennes, Rennes, France

**Keywords:** Transcatheter Aortic Valve Implantation, Cusp-overlap, Conduction disturbances, Implantation depth, Membranous Septum

## Abstract

New-onset conduction disturbances (NOCD) including high-degree atrioventricular block (HAVB), permanent pacemaker implantation (PPI) and new-onset left bundle branch block after transcatheter aortic valve implantation (TAVI) remain common and are influenced by implantation depth (ID) relative to patient-specific anatomy, particularly membranous septum length.To compare the cusp-overlap (COL) and conventional three-cusp coplanar (TCC) projection techniques with a focus on ID relative to membranous septum length and its association with conduction outcomes. A total of 501 patients undergoing TAVI with pre-procedural multidetector computed tomography were included. The primary endpoint was a composite of HAVB or PPI at 30 days. ID was measured on the final angiography. Propensity score overlap weighting analysis was performed to assess the effects of COL on ID and the main outcome.COL was significantly associated with higher valve implantation (ID 4.0 ± 1.95 mm vs. 5.54 ± 2.6 mm; β coefficient = -1.76; 95% CI: -2.24 to -1.26). The primary endpoint occurred at similar rates in both groups (15% vs. 19%; *p* = 0.24), and COL was not independently associated with the primary outcome (OR 0.63, 95% CI 0.36–1.12). Deeper implantation was associated with the primary outcome (4.66 ± 2.33 mm vs. 6.56 ± 2.66 mm; *p* < 0.01). A greater difference between membranous septum length and ID independently predicted lower event risk.COL was not independently associated with reduced NOCD but may facilitate higher implantation relative to membranous septum length, supporting an individualized, anatomy-guided implantation strategy.

**Graphical Abstract:**

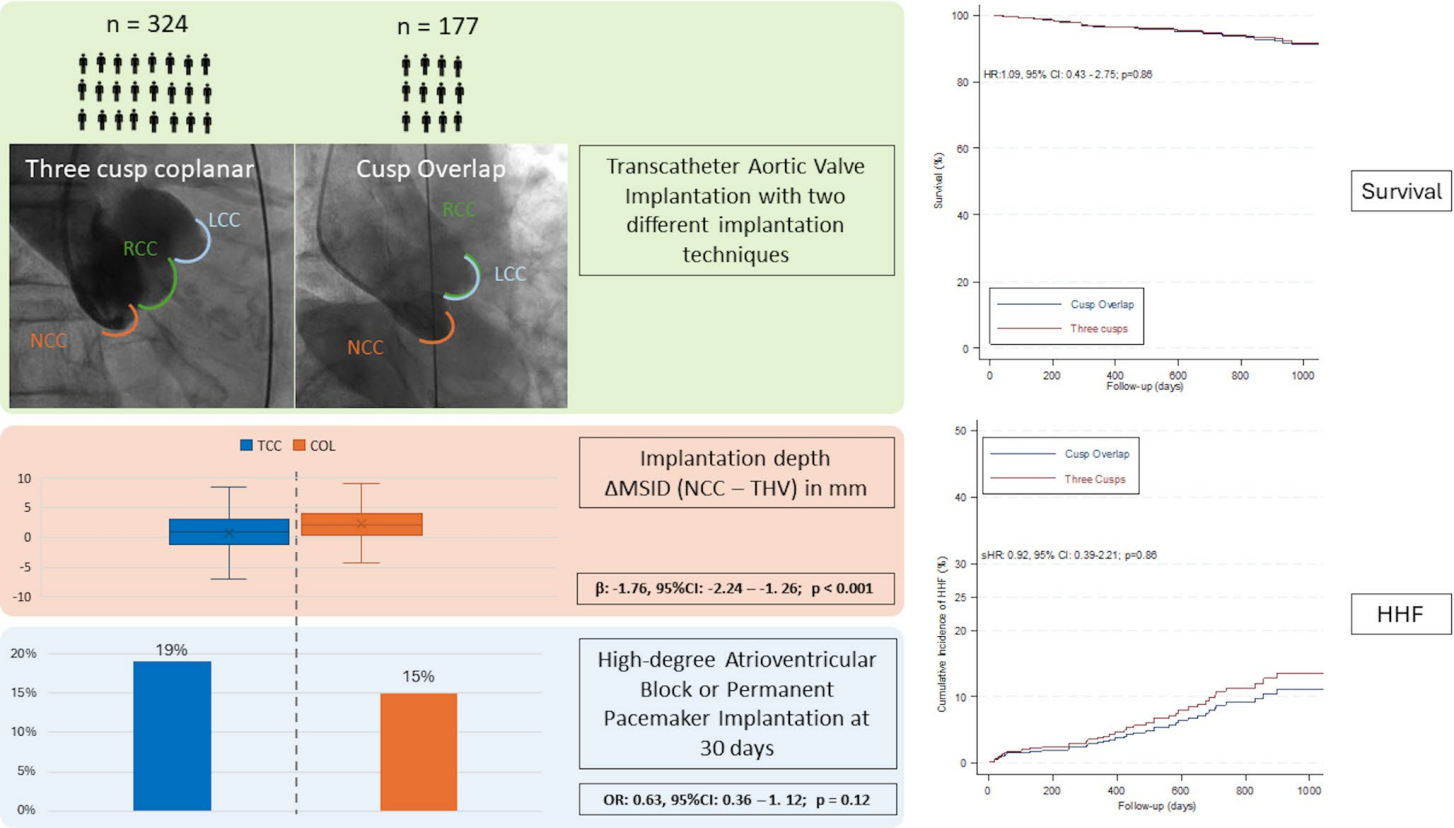

**Supplementary Information:**

The online version contains supplementary material available at 10.1007/s12928-026-01276-0.

## Introduction

Since the inception of transcatheter aortic valve implantation (TAVI) approximately twenty years ago [1], technological enhancements and procedural simplifications have allowed its routine use in current practice to treat symptomatic aortic valve stenosis [2, 3] New-onset conduction disturbances (NOCD), including high-degree atrioventricular block (HAVB), permanent pacemaker implantation (PPI), and new-onset persistent left bundle branch block (NOP-LBBB), remain common complications following transcatheter aortic valve implantation (TAVI) and are associated with poorer long-term outcomes [4–8]. As TAVI indications expand to younger and lower-risk populations, strategies to mitigate conduction disturbances have gained increasing importance.

Beyond patient-related and anatomical predictors, increased attention has recently been paid to per-procedural methods for reducing NOCD. The cusp-overlap (COL) technique has been described as an alternative to conventional three-cusps coplanar (TCC) projection, aiming to reduce parallax and improve visualization of the non-coronary cusp, thereby facilitating more predictable valve implantation depth (ID) [9]. According to recent works, higher implantation of transcatheter heart valve (THV) decreases the rate of NOCD requiring PPI for balloon-expandable valve (BEV) [10] and self-expandable valve (SEV) [11–15]. The role of ID in NOCD is closely related to inter-individual anatomical variations like membranous septum length (MSL) [16, 17] and the volumes and distribution of calcifications of the aortic valve complex [18, 19], which were not systematically accounted for in recent observational studies. Most studies comparing the COL and TCC techniques involved a “historical” cohort of TCC, which also raises the problem of heterogeneous management of NOCD for which guidelines were only recently published [20, 21]. We therefore hypothesized that COL does not exert a direct protective effect on NOCD per se, but rather may indirectly influence outcomes by facilitating higher implantation relative to patient-specific anatomy, quantified by the difference between MSL and ID (ΔMSID).

Accordingly, the present study aimed to compare COL and TCC techniques in a contemporary cohort, with a specific focus on the mechanistic role of implantation depth relative to membranous septum length.

## Methods

### Study population and TAVI procedures

All patients who underwent a multidetector computed tomography (MDCT) in the University Hospital of Rennes before the implantation of a THV were screened for this study. Patients with previous PPI, valve-in-valve procedures, and unmeasurable ID due to excessive parallax or lack of contrast enhancement in the final angiogram were excluded from the final analysis. Procedures were performed after discussion by a multidisciplinary Heart Team between January 2020 and December 2023 via the femoral or carotid accesses in our center. The choice of implantation projection (COL or TCC) was left to operator discretion, without predefined anatomical or procedural criteria.

Every patient had a 12-lead electrocardiogram (ECG) before and daily after the procedure. They underwent continuous telemetry monitoring for at least twenty-four hours. NOCD were defined according to the American Heart Association, American College of Cardiology Foundation, and Heart Rhythm Society recommendations to standardize and interpret the electrocardiogram [22]. Their management was based on JACC scientific expert consensus [20] from the inclusion, followed by the European Society of Cardiology’s guidelines on cardiac pacing from 2021^21^. For patients with milder new-onset or worsening conduction disturbances persisting without improvement upon serial ECG at least 48 h after the procedure, PPI was guided by an electrophysiological study [23]. Clinical and echocardiographic characteristics before and after TAVI were also reported. All patients gave written informed consent for the procedures and anonymous collection of their data, which were prospectively gathered in an electronic database as part of the national registry [24]. This work was approved by the institutional review board and was conducted following the provisions of the Declaration of Helsinki.

## Multidetector computed tomography

**Fig. 1:**
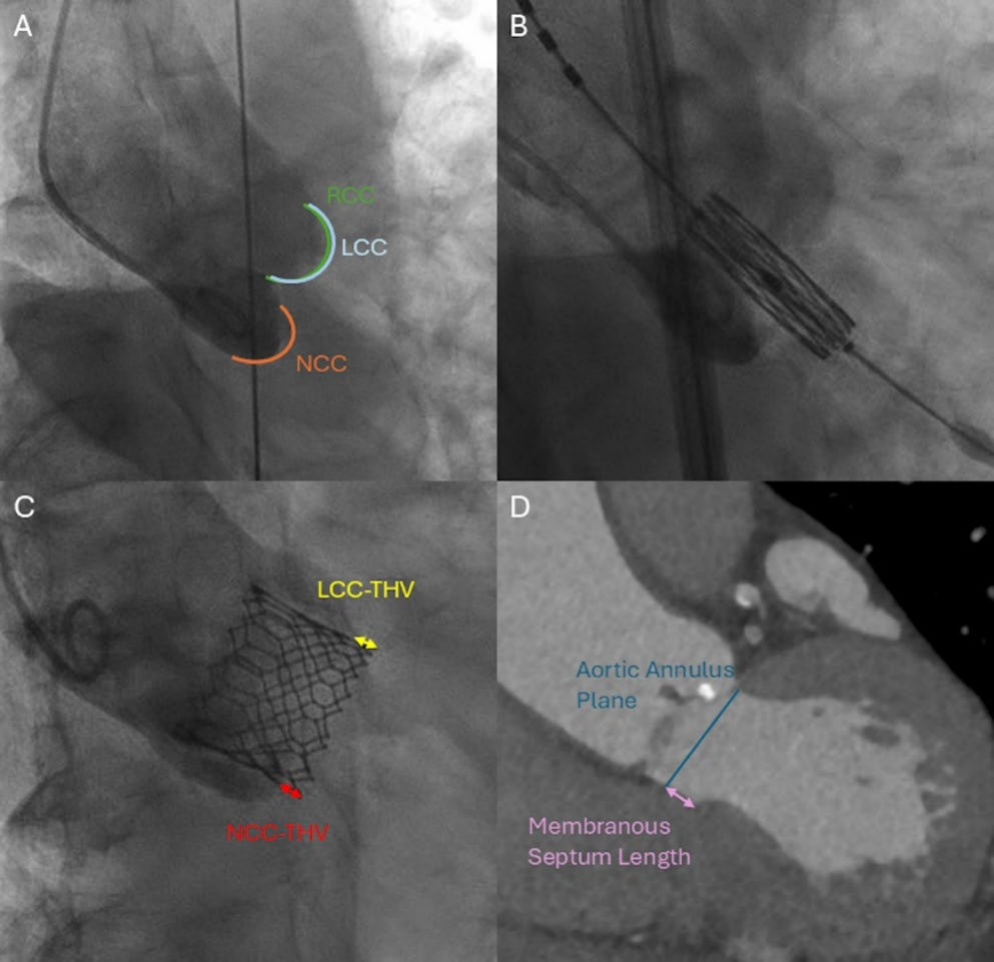
Illustrations of the cusp-overlap technique and the assessment of implantation depth and membranous septum length Cusp-overlap view using right anterior oblique and caudal incidence, superimposes the right coronary-cusp (RCC) and the left coronary-cusp (LCC) (**A**). Positioning a SAPIEN 3 valve using the cusp-overlap view to assess implantation depth (**B**). Implantation Depth measurement from the nadir of the non-coronary cusp (NCC) and the LCC cusps to the inferior edge of the transcatheter heart valve (**C**). Membranous septum length measurement from the aortic annulus plane to the lower part of the membranous septum (**D**). ΔMSID was calculated as the difference between membranous septum length and implantation depth for the left and non-coronary cusp.

Enhanced-contrast electrocardiographically gated MDCTs were performed at end-systole (≈ 30% of the heart cycle) and acquired with collimation, as recommended for measuring aortic valve complex dimensions and peripheral vascular access [25, 26]. Working projections, MSL, aortic annulus areas, and diameters were manually obtained and reported for each patient during postprocessing from the workstation AW Server 3.2 (General Electric Healthcare). Measurements were performed retrospectively by a single experienced operator. The aortic annulus was defined by the luminal contour within a virtual plane aligned with the basal attachment points of the three aortic valve cusps [25]. For a standardized analysis of the membranous septum, the cursor in the perpendicular co-planar view was placed on the intersect of the non-coronary and the right coronary cusp. The coronal view was swept to find the lower part of the membranous septum [25]. Its length was the Euclidean distance between its lower part and the aortic annulus plane. Intra-observer variability assessment was performed on 50 randomly selected cases (Fig. 1**in the Supplemental Appendix)**.

**Fig. 2:**
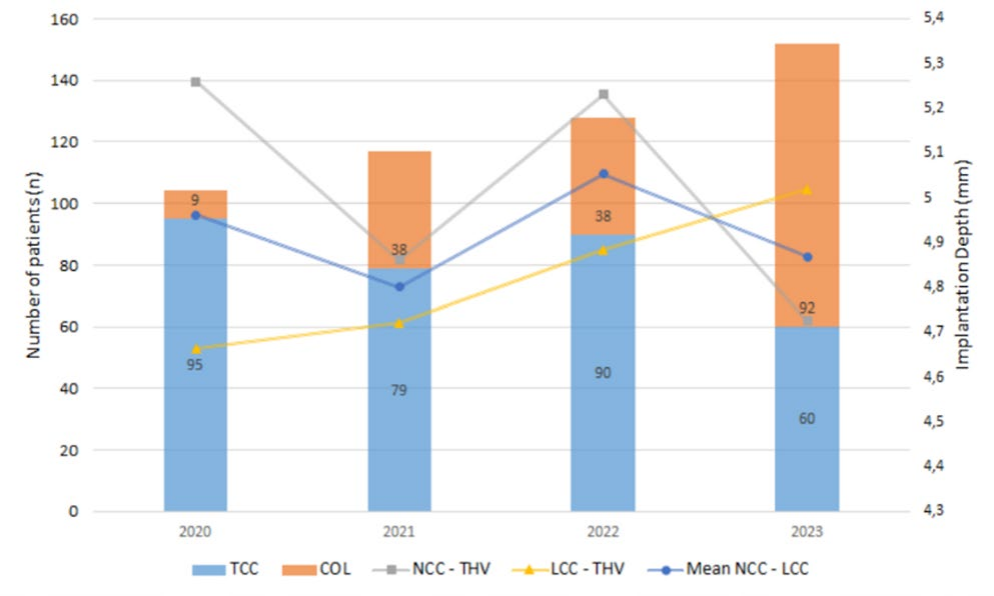
Temporal trends of implantation techniques and implantation depth COL: Cusp-Overlap; LCC: Left Coronary Cusp; NCC: Non-Coronary Cusp; TCC: Three Cusps Coplanar; THV: Transcatheter Heart Valve

Volumes of calcifications were obtained from a fully automated detection of landmarks within the aortic valve complex [27], which was separated into three sections in the craniocaudal axis. The device landing zone (DLZ) was defined as the cylinder ranging from 3 mm above to 2 mm below the aortic annulus plane. The left ventricular outflow tract (LVOT) was defined as the 10 mm below the aortic annulus plane along the centerline. The inferior limit of the total leaflet (TL) sector was the plane of the annulus aortic plane. We used an arbitrary height of 12 mm for this sector. The upper leaflet (UL) section was limited below by the DLZ and has the same upper limit as the TL section. Each craniocaudal section was subdivided into three sub-sectors corresponding to the projections of the NCC, the LCC and the RCC [18]. Overall, 17 sectors were individualized for the analysis (Fig. 2**in the Supplemental Appendix**). A threshold of 850 Hounsfield-Unity (HU) [28] was used to quantify the volumes of calcifications, with a systematic review by an expert operator to detect any artifact that could lead to a misestimation. Each volume was expressed in mm^3^ of calcifications and as a percentage of the total sector volume.

## Implantation depth measurement

The ID was retrospectively measured on the final angiogram using the software Telemis (Version 4.96). The depth was measured from the inferior edge of the THV to the nadir of the noncoronary cusp (NCC) expressed by NCC-THV and left-coronary cusp (LCC) expressed by LCC-THV (Fig. 1). Mean LCC – NCC corresponds to the arithmetic mean between LCC-THV and NCC-THV. Finally, we defined ∆MSID-LCC and ∆MSID-NCC as the differences between the MSL and the ID calculated from the LCC and the NCC, respectively. ∆MSID Mean LCC – NCC was the difference between MSL and the arithmetic mean of LCC-THV and NCC-THV (Fig. 1).

## Outcomes

The primary outcome of this study was the composite of high-degree atrio-ventricular block (HAVB) or PPI at 30 days. The secondary outcomes were NOP-LBBB, and the ID expressed as NCC-THV as the objective of the COL technique is theoretically to achieve a lower ID below the NCC. Hospitalizations for heart failure (HHF), PPI during the follow-up, and death from any cause were also reported for the analysis.

### Statistical analysis

Continuous variables were expressed as mean ± standard deviation (SD), or median and interquartile range [Q25-75]; while categorical variables as frequencies followed by percentages. Normal distribution was tested using the Kolmogorov-Smirnov test for each variable. Qualitative data were compared using the χ2 or Fisher exact tests while quantitative data were compared using t-tests or the Mann–Whitney U tests. The evolution of the use of the COL view and its impact on ID throughout the study was assessed using the Kruskal-Wallis test. To reduce the bias inherent to the non-randomized nature of the study in the analysis of the treatment effect of being treated using the COL technique on the primary and secondary outcomes, we applied propensity-score overlap weighting to generate two study populations with comparable baseline characteristics [29]. The propensity-score of being treated using the COL technique was developed using a logistic regression model including the following variables: age, sex, height, weight, hypertension, diabetes mellitus, previous CAD, previous valvular intervention, atrial fibrillation, chronic pulmonary disease, chronic kidney disease, EuroScore II, bicuspid aortic valve, annulus area, percentage of oversizing relative to the annulus area, membranous septum length, total aortic valve calcification volume, NC-DLZ calcification volume, baseline first-degree atrioventricular block, baseline right bundle branch block, valve type, pre- and post-dilation, and year of TAVI. The balance between groups was evaluated by analyzing the standardized differences before and after weighting. A post-weighting difference < 0.1 was considered as an optimal bias reduction. Linear and logistic regressions were used to analyze the effect of being treated using the COL on continuous and binary outcomes, respectively. Secondary confirmatory analyses were performed using an inverse probability of treatment weighting with the above-described propensity score and using multivariable regressions. For the latter, variables associated with the outcome in univariable analysis at a p-value of < 0.1 were included in the multivariable model. To avoid multicollinearity the tolerance and variance inflation factor as well as bivariate correlations were assessed for each multivariable model. Long-term outcomes were analyzed using Cox and competing-risk regression adjusted for the propensity score and pre-defined variables likely to be associated with the outcomes based on current literature: left ventricular ejection fraction at discharge, at least mild (2+/4+) aortic regurgitation post-TAVI, and the occurrence of HAVB or PPI within 30 days of TAVI. The intra-observer variability of the measurement of the membranous septum length was performed by Bland-Altman’s analysis and Lin’s Concordance Coefficient. A p-value < 0.05 was used for all statistical tests to indicate statistical significance. Statistical analysis was performed with the use of Statistical Package for Social Sciences version 25 (SPSS, Chicago, IL, USA), and Stata Statistical Software release 13 (StataCorp, LLC, College Station, TX, USA).

## Results

### Baseline characteristics

During the inclusion period, we screened 596 patients with MDCT whose indication of TAVI was retained. Among them, 42 had previous PPI or implantable cardioverter defibrillator, 8 had “valve-in-valve” procedures, and 41 had excessive parallax or unmeasurable ID measurements on the final angiogram. There were no cases of conversion to open heart surgery, THV embolization, or coronary occlusion. Finally, 501 patients were enrolled in the final analysis (**supplemental Fig. 3**), including 233 women (46.5%), with a mean age of 80.3 ± 7.4 years and a Euroscore II of 11.2 ± 9.1% (Table 1). Femoral access was used in 447 procedures and left carotid access in 54, with 379 patients (75.65%) receiving a BEV (SAPIEN 3, Edwards Lifescience) and 122 a SEV (24.35%) encompassing 67 (13.37%) EvolutR/Pro (Medtronic), 39 (7.78%) Portico/Navitor (Abbott), and 15 (2.99%) Acurate Neo (Boston Scientific). Regarding the pre-procedural conduction disturbances, 47 patients (9.4%) presented a right bundle branch block (RBBB), 128 (30.8% of patients without atrial arrhythmia) had a first-degree atrio-ventricular block (FD-AVB). A total of 85 patients (16.97%) had atrial arrhythmia. The mean aortic gradient was 55.8 ± 17.21 mmHg, and the indexed aortic valve area was 0.41 ± 0.11 cm²/m². The mean MSL was 6.32 ± 2.10 mm. The two groups were comparable regarding baseline conduction disturbances, calcification rates and echocardiographic parameters. The repartition of the MSL is reported in Fig. 4**in the Supplemental Appendix**.

**Table 1:** Baseline characteristics of the study population

	General Population (*n* = 501)	Three Cusp Group (*n* = 324)	Cusp-Overlap Group (*n* = 177)	*p*-value
Clinical Characteristics				
Age, years	80.3 (7.4)	80.3 (6.65)	80.4 (8.59)	0.93
Female, n	233 (46.5)	132 (41%)	101 (57%)	**< 0.01**
Weight, kg	73.1 (15.8)	74.6 (16.5)	70.3 (14.1)	**< 0.01**
Height, m	1.64 (0.08)	1.65 (0.08)	1.63 (0.08)	0.13
BMI, kg/m²	27.5 (5.1)	27.5 (5.35)	26.3 (4.50)	**< 0.01**
Euroscore II Logistic, %	11.2 (9.1)	10.9 (8.72)	11.8 (9.71)	0.27
Hypertension, n	375 (75%)	248 (77%)	127 (72%)	0.24
Diabetes mellitus, n	98 (20%)	74 (23%)	24 (14%)	**0.01**
Previous CAD, n	225 (45%)	153 (47%)	72 (41%)	0.12
Previous valvular intervention, n	10 (2%)	5 (1.5%)	5 (2.8%)	0.33
Creatinine, µmol/L	93.8 (60.8)	97.5 (71.4)	87.1 (33.1)	0.03
Chronic Renal Disease, n	144 (29%)	89 (27%)	55 (31%)	0.39
Chronic Pulmonary disease, n	52 (10%)	33 (10%)	19 (11%)	0.85
***Baseline ECG***				
Atrial Fibrillation, n	85 (17%)	53 (16%)	32 (18%)	0.62
PR interval (ms)	196 (42.1)	188 (37.8)	184 (39.2)	0.43
FDAVB, n	128 (31%)	88 (32%)	40 (28%)	0.30
RBBB, n	47 (9.4%)	32 (9.9%)	15 (8.5%)	0.61
LAHB, n	79 (16%)	59 (18%)	20 (11%)	0.04
LBBB, n	38 (7.6%)	22 (6.8%)	16 (9%)	0.36
***MDCT data***				
Membranous Septum Length, mm	6.32 (2.10)	6.38 (2.19)	6.21 (1.92)	0.39
Annulus Area, mm²	434 (88.0)	441 (85.7)	421 (90.8)	**0.02**
Annulus Diameter, mm	23.3 (2.37)	23.5 (2.32)	22.9 (2.42)	**0.01**
Bicuspid Aortic Valve, n	10 (2%)	8 (2.5%)	2 (1.1%)	0.31
Total volumes of calcifications, mm3	412 (327)	410 (305)	417 (366)	0.81
%	4.27 (3.40)	4.06 (3.35)	4.67 (3.47)	0.06
DLZ, mm3	50.6 (56.4)	50.0 (54.7)	51.5 (59.6)	0.79
%	1.61 (1.73)	1.58 (1.75)	1.67 (1.69)	0.59
DLZ-LCC	14.8 (25.4)	14.6 (24.5)	15.3 (27.2)	0.78
%	1.51 (2.48)	1.48 (2.49)	1.56 (2.47)	0.73
DLZ-NCC	21.3 (30.1)	20.6 (27.8)	22.5 (33.9)	0.53
%	2.07 (2.81)	1.99 (2.69)	2.23 (3.02)	0.38
DLZ-RCC	14.5 (23.7)	14.9 (25.7)	13.7 (19.6)	0.58
%	1.27 (2.09)	1.27 (2.21)	1.27 (1.85)	1.00
LVOT, mm3	26.7 (62.5)	24.1 (49.1)	31.5 (81.5)	0.28
%	0.431 (1.01)	0.395 (0.91)	0.497 (1.18)	0.32
LVOT-LCC	10.7 (36.9)	8.90 (25.6)	14.1 (51.4)	0.21
%	0.548 (1.77)	0.469 (1.41)	0.692 (2.28)	0.24
LVOT-NCC	12.6 (33.9)	11.7 (27.7)	14.1 (43.1)	0.50
%	0.621 (1.68)	0.591 (1.54)	0.676 (1.93)	0.62
LVOT-RCC	3.44 (12.6)	3.54 (11.8)	3.26 (13.8)	0.82
%	0.156 (0.6)	0.164 (0.6)	0.141 (0.6)	0.68
Total Leaflet, mm3	386 (300)	385 (283)	386 (330)	0.99
%	5.01 (3.47)	4.95 (3.26)	5.12 (3.84)	0.61
TL-LCC	102 (103)	102 (94.4)	103 (117)	0.87
%	4.19 (3.78)	4.14 (3.54)	4.29 (4.21)	0.70
TL-NCC	164 (134)	163 (130)	168 (142)	0.71
%	6.58 (4.71)	6.43 (4.55)	6.86 (4.98)	0.34
TL-RCC	119 (114)	121 (110)	115 (120)	0.58
%	4.26 (3.83)	4.29 (3.64)	4.21 (4.16)	0.84
Upper Leaflet, mm3	344 (275)	345 (260)	344 (301)	0.98
%	5.94 (4.24)	5.88 (3.97)	6.07 (4.70)	0.65
UL-LCC	90.9 (94.7)	90.3 (87.1)	91.9 (108)	0.86
%	4.94 (4.67)	4.88 (4.32)	5.06 (5.25)	0.70
UL-NCC	147 (124)	146 (122)	149 (129)	0.77
%	7.83 (5.85)	7.66 (5.71)	8.14 (6.11)	0.40
UL-RCC	106 (105)	108 (100)	103 (113)	0.58
%	5.07 (4.71)	5.11 (4.43)	4.98 (5.19)	0.77
***Echocardiographic Parameters***				
Mean Gradient, mmHg	55.8 (17.2)	55.7 (17.0)	56.2 (17.7)	0.76
Peak aortic velocity, m/s	4.59 (0.69)	4.59 (0.68)	4.60 (0.72)	0.91
Indexed Surface, cm²/m²	0.409 (0.11)	0.411 (0.12)	0.404 (0.1)	0.48
LVEF, %	61.9 (11.4)	61.7 (11.2)	62.2 (11.9)	0.66
Indexed LVEDD, mm/m²	25.8 (4.27)	25.8 (4.39)	25.8 (4.07)	0.86
Low Flow/Low Gradient, n	34 (6.9%)	19 (5.9%)	15 (8.5%)	0.28

BMI, Body Mass Index; CAD, Coronary Artery Disease; DLZ, Device Landing Zone; ECG, Electrocardiogram; FD-AVB, First Degree Atrio-Ventricular Block; LAHB, Left Anterior Hemi-Block; LBBB, Left Bundle Branch Block; LCC, Left Coronary Cusp; LVEF, Left Ventricular Ejection Fraction; LVEDD, Left Ventricular end-diastolic Diameter; LVOT, Left Ventricular Outflow Tract; MDCT, Multidetector Computed Tomography; NCC, Non Coronary Cusp; RCC, Right Coronary Cusp; UL, Upper Leaflet

The COL technique was used in 177 patients (35.3%); whereas 324 patients (64.7%) were treated using the conventional TCC technique. The utilization of the COL in current practice increased compared to the TCC technique, from 8.6% in 2020 to 60.5% in 2023 (*p* < 0.01). Nevertheless, this change of practice did not lead to significantly shorter ID for NCC-THV (*p* = 0.53 in the COL group and *p* = 0.92 in the TCC group), LCC-THV (*p* = 0.31 in the COL group and *p* = 0.13 in the TCC group), or mean LCC-NCC (*p* = 0.26 in the COL group and *p* = 0.39 in the TCC group) over time. All data regarding the evolution of techniques’ use and ID are illustrated in Fig. 2. Table 2 exposes procedural and post-procedural data. Predilatation was performed in 110 (62%) for the COL group vs. 152 (47%) patients in the TCC group (*p* < 0.01), and THV used in the COL group were more frequently SEV (33.9%) than in the TCC group (19.1%) (*p* < 0.01).

**Table 2:** Procedural and post-procedural data of the study population

Femoral Access, *n*	General Population (*n* = 501)	Three Cusp Group (*n* = 324)	Cusp-Overlap Group (*n* = 177)	*p*-value
	447 (89%)	283 (87%)	164 (93%)	0.07
Carotid Access, n	54 (11%)	41 (13%)	13 (7.3%)	0.07
Predilatation, n	262 (52%)	152 (47%)	110 (62%)	**< 0.01**
Post-dilatation, n	43 (8.6%)	24 (7.4%)	19 (11%)	0.2
***THV***				
BEV, n	379 (75.6%)	262 (80.1%)	117 (66.1%)	**< 0.01**
SEV, n	122 (24.4%)	62 (19.1%)	60 (33.9%)	**< 0.01**
Edwards, n	379 (76%)	262 (81%)	117 (66%)	**< 0.01**
CoreValve, n	67 (13%)	31 (9.6%)	36 (20%)	**< 0.01**
Portico, n	13 (2.6%)	9 (2.8%)	4 (2.3%)	1
Acurate NEO, n	15 (3%)	14 (4.3%)	1 (0.56%)	**0.02**
Navitor, n	26 (5.2%)	7 (2.2%)	19 (11%)	**< 0.01**
***THV Size***				
20, n	8 (1.6%)	5 (1.5%)	3 (1.7%)	1.00
23, n	153 (31%)	93 (29%)	60 (34%)	0.23
25, n	20 (4%)	15 (4.6%)	5 (2.8%)	0.32
26, n	183 (37%)	126 (39%)	57 (32%)	0.14
27, n	15 (3%)	8 (2.5%)	7 (4%)	0.35
29, n	114 (23%)	72 (22%)	42 (24%)	0.70
34, n	8 (1.6%)	5 (1.5%)	3 (1.7%)	1.00
**Area oversizing**,** %**	12.7 (1.1–27.3)	11.6 (0.9–25.2)	16.7 (2.4–32.2)	**0.007**
***Depth Implantation***				
LCC-THV, mm	4.84 (2.29)	5.08 (2.39)	4.40 (2.02)	**< 0.01**
Mean NCC – LCC, mm	4.92 (2.14)	5.32 (2.23)	4.19 (1.76)	**< 0.01**
NCC-THV, mm	5.00 (2.50)	5.54 (2.60)	4.00 (1.95)	**< 0.01**
∆MSID-LCC, mm	1.46 (3.18)	1.27 (3.32)	1.82 (2.90)	0.06
∆MSID mean LCC – NCC, mm	1.40 (3.08)	1.05 (3.20)	2.03 (2.75)	**< 0.01**
∆MSID-NCC, mm	1.32 (3.32)	0.833 (3.44)	2.22 (2.91)	**< 0.01**
***New Onset Conduction Disturbances***				
PPI at 30 days, n	90 (18.0%)	63 (19%)	27 (15%)	0.24
PPI at discharge, n	85 (17.0%)	60 (19%)	25 (14%)	0.21
Complete AVB, n	51 (10.2%)	33 (10%)	18 (10%)	0.99
New-Onset FD-AVB, n	57 (11.4%)	39 (16%)	18 (14%)	0.64
PR interval, ms	196.2 (42.0)	198 (42.9)	193 (40.4)	0.22
New-Onset LBBB, n	105 (20.1%)	74 (25%)	41 (26%)	0.92
Prognostic NOCD, n	168 (33.5%)	108 (33%)	60 (34%)	0.90
***Echocardiographic Parameters***				
Mean Gradient, mmHg	11.9 (5.18)	12.4 (5.39)	10.9 (4.63)	**< 0.01**
Peak aortic velocity, m/s	2.23 (0.470)	2.29 (0.469)	2.12 (0.451)	**< 0.01**
LEVF, %	62.5 (9.76)	62.0 (9.58)	63.5 (10.0)	0.10
Indexed aortic area, mm/m²	1.18 (0.420)	1.13 (0.397)	1.27 (0.446)	**< 0.01**
***Follow-up***				
Follow-up, months	17.6 (12.0)	19.4 (12.3)	13.9 (10.3)	**< 0.01**
Rehospitalization for HF, n	43 (10%)	36 (12.6%)	7 (4.9%)	**0.01**
PPI > 30 days	12 (2.4%)	11 (3.4%)	1 (0.6%)	**< 0.05**
Death, n	35 (7.9)	30 (10.1%)	5 (3.4%)	**0.01**

AVB, Atrio-Ventricular Block; BEV, Balloon Expandable Valve; COL, Cups-Overlap; ∆MSID, difference between Membranous Septum length and Implantation Depth; FDAVB, First-Degree Atrio-Ventricular Block; HF, Heart Failure; LBBB, Left Bundle Branch Block; LCC, Left Coronary Cusp; LVEF, Left Ventricular Ejection Fraction; NOCD, New-Onset Conduction Disturbances; NCC, Non Coronary Cusp; PPI, Permanent Pacemaker Implantation; RCC, Right Coronary Cusp; THV, Transcatheter Heart Valve.

## New-onset conduction disturbances

**Fig. 3:**
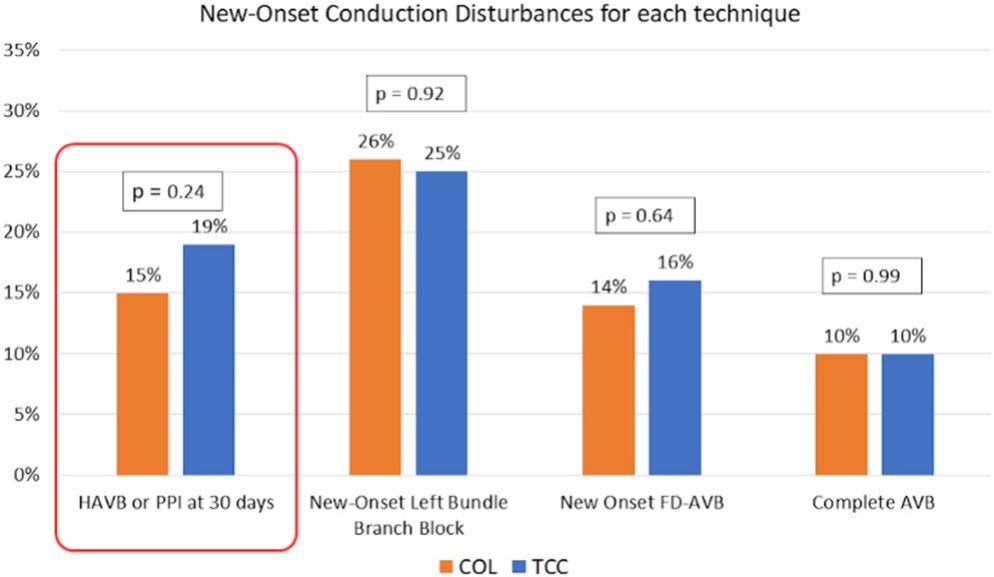
Rates of New-Onset Conduction Disturbances for each implantation technique AVB: atrioventricular block; COL: cusp-overlap; FD-AVB: first degree atrioventricular block; HAVB: High-degree atrioventricular block; PPI: Permanent pacemaker implantation; TCC: Three cusps coplanar

Ninety patients (18.0%) had HAVB or PPI in the first 30 days, including 5 (1.0%) after hospital discharge. Ninety-six patients (19.2%) presented a NOP-LBBB without PPI at 30 days. The primary outcome occurred in 27 patients (15%) in the COL group and 63 patients (19%) in the TCC group (*p* = 0.24) (**Central Illustration**). There was also no difference between the two groups regarding the occurrence of NOP-LBBB (26% vs. 25%, *p* = 0.92). Rates of NOCD are summarized in Fig. 3.

Higher ∆MSID was significantly associated with a lower rate of NOCD: ∆MSID-NCC was − 0.8 ± 3.6 mm in the primary outcome group, compared to 1.8 ± 3.1 mm (*p* < 0.01). ∆MSID-LCC was − 0.3 ± 3.5 vs. 1.8 ± 3.0 mm (*p* < 0.01) and ∆MSID-Mean (LCC-NCC) was − 0.5 ± 3.4 vs. 1.8 ± 2.8 mm (*p* < 0.01), respectively. Comparisons between groups with or without the primary outcome are shown in **Supplemental Tables 1 and 2**. Relationships between the primary outcome and the ID parameters are displayed in **Supplemental Fig. 5**.

### Implantation depth

COL was associated with a higher valve implantation: 5.54 ± 2.60 vs. 4.00 ± 1.95 mm for NCC-THV (*p* < 0.01); 5.08 ± 2.39 vs. 4.40 ± 2.02 mm for LCC-THV (*p* < 0.01); and 5.32 ± 2.23 vs. 4.19 ± 1.76 mm for Mean LCC-NCC (*p* < 0.01). This resulted in a greater ∆MSID relative to the NCC: 2.2 ± 2.9 vs. 0.8 ± 3.4 mm (*p* < 0.01). All data on the relationship between ID and the implantation technique used are illustrated in **Supplemental Fig. 5**.

### Adjusted analyses

**Fig. 4:**
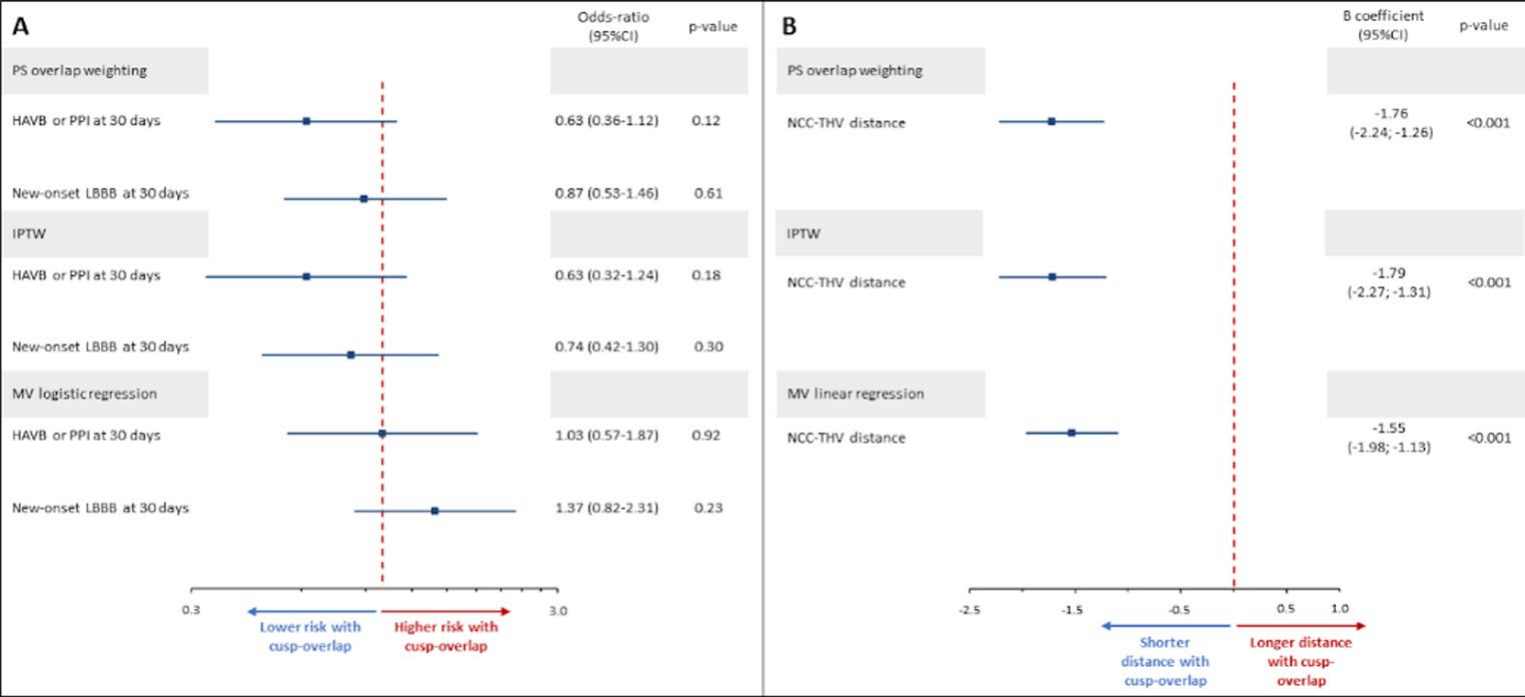
Adjusted associations between the implantation technique and the primary and secondary outcomes Panel A – association with new-onset conduction disturbances. Panel B – association with implantation depth. CI, Confidence Interval; HAVB, High-degree Atrioventricular Block; IPTW, inverse-probability-of-treatment weighting; LBBB, Left Bundle Branch Block; MV, Multivariable Linear; NCC, Non Coronary-Cusp; PPI, Permanent Pacemaker Implantation; PS, Propensity Score; THV, Transcatheter Heart Valve. Multivariable logistic regression for HAVB/PPI was adjusted for: age, Logistic Euroscore II, Total aortic valve calcification volume, baseline first-degree AV block, baseline RBBB, Valve type, Delta membranous septum length - NCC to THV distance. Multivariable logistic regression for new-onset LBBB was adjusted for NC-DLZ calcification volume, indexed LVEDD, TTE LVOT diameter, NCC to THV distance and valve type. Multivariable linear regression for NCC to THV distance was adjusted for hypertension, chronic pulmonary disease, annulus area on MDCT, femoral access, BEV (vs. all SEV), and baseline LVEF

Figure 6**in the Supplemental Appendix** exposes standardized differences before and after propensity score overlap weighting. An optimal balance of baseline variables was observed after weighting. COL was not independently associated with a reduction of the primary outcome (Fig. 4A): OR (95%CI) = 0.63 (0.36–1.12, *p* = 0.12); or with the occurrence of NOP-LBBB: OR (95%CI) = 0.87 (0.53–1.46, *p* = 0.61). Nevertheless, COL was significantly associated with a lower NCC-THV distance (β= −1.76, 95%CI: −2.24- −1.26; *p* < 0.001) (Fig. 4B). Results were consistent in inverse probability of treatment weighting and multivariable analyses. ΔMSID-NCC emerged as an independent anatomical predictor of the primary endpoint in multivariable analysis (OR = 0.78, 95% CI: 0.71–0.85; *p* < 0.001). Other multivariable predictors of the primary outcome included age (OR = 1.06, 95% CI: 1.01–1.11; *p* = 0.02), baseline FD-AVB (OR = 3.28, 95% CI: 1.88–5.75; *p* < 0.001), baseline RBBB (OR = 7.84, 95% CI: 3.75–16.4; *p* < 0.001), implantation of a Portico/Navitor prosthesis (OR = 3.75, 95% CI: 1.60–8.82; *p* = 0.002).

### Follow-up

**Fig. 5:**
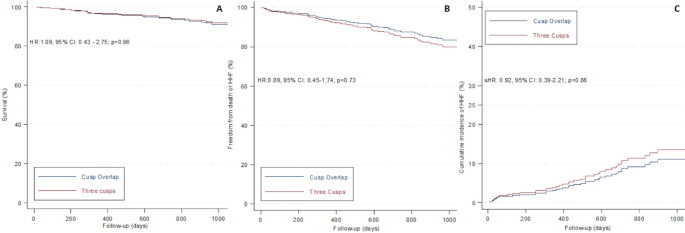
Outcomes at long-term follow-up according to the implantation technique.Survival (A), survival free from heart failure hospitalization (B), and cumulative incidence of heart failure hospitalization (C) CI: Confidence interval; HHF: Heart failure hospitalization; HR: Hazard ratio, sHR: sub-hazard ratio

**Fig. 6:**
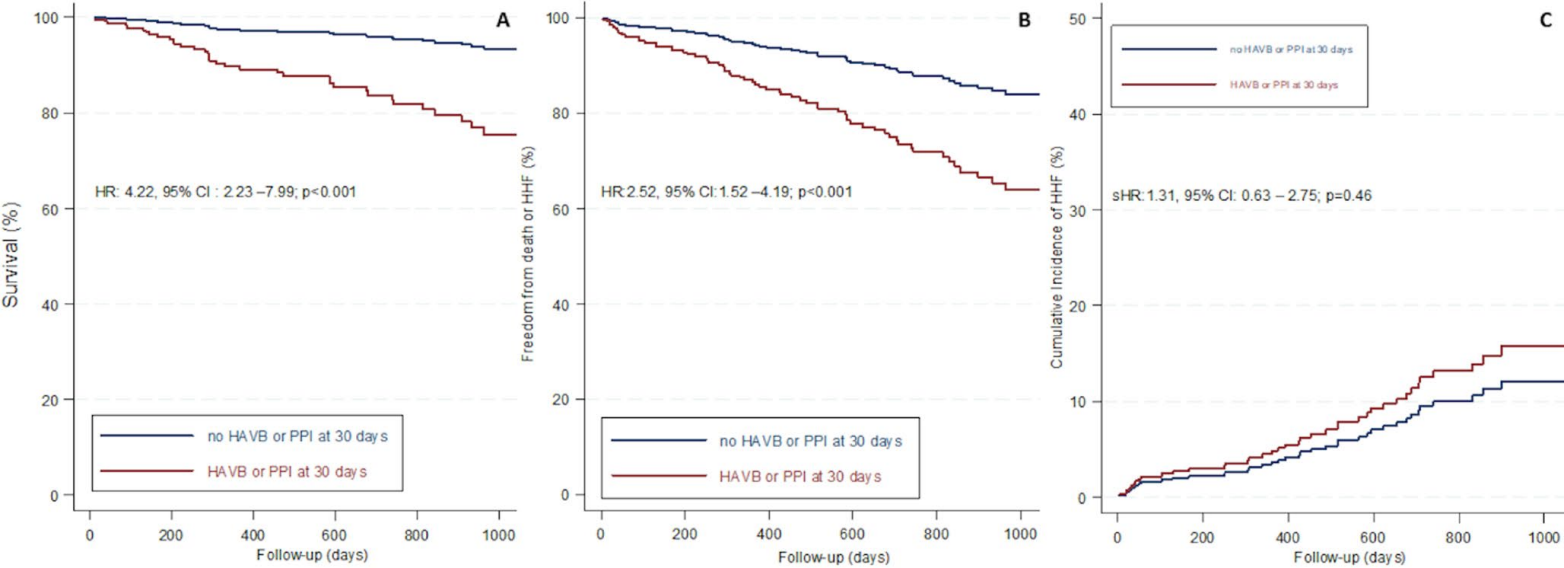
Outcomes at long-term follow-up according to the occurrence of the primary outcome Survival (**A**), survival free from heart failure hospitalization (**B**), and cumulative incidence of heart failure hospitalization (**C**). Abbreviations as in Fig. 5.

At a median follow-up of 525 (310–843) days, 41 patients (8.2%) died, including 34 (10.5%) in the TCC group and 7 (4.0%) in the COL group (*p* = 0.01). HHF occurred in 47 patients (9.4%) of whom 37 patients (11.5%) were treated using the TCC technique and 10 patients (5.6%) using the COL technique (*p* = 0.03). However, in multivariable analyses, the COL technique was not significantly associated with all-cause mortality, HHF, or the composite of those outcomes (Fig. 5). On the contrary, the occurrence of HAVB or PPI within 30 days of TAVI was significantly associated with a higher risk of all-cause mortality and the composite of all-cause mortality or HHF (Fig. 6). PPI was required beyond 30 days of the TAVI procedure in 12 patients (3.7%) of the TCC group and 2 patients (1.1%) of the COL group (*p* = 0.15).

## Discussion

In this contemporary cohort, the cusp-overlap technique was not independently associated with a reduction in HAVB or PPI at 30 days. Our findings therefore primarily provide confirmatory evidence that implantation depth relative to membranous septum length, rather than the angiographic projection itself, is a key determinant of conduction outcomes after TAVI. Nonetheless, COL significantly reduced ID, thus the COL technique should be interpreted as a facilitating imaging tool that may support anatomy-guided implantation strategies, rather than as a direct determinant of conduction outcomes. Importantly, the projection strategy itself was not independently associated with long-term outcomes after multivariable adjustment. In contrast, early conduction disturbances were strongly associated with subsequent mortality and heart failure hospitalization, underscoring that conduction injury rather than implantation projection is among the key drivers of late clinical events.

The COL uses right anterior oblique and caudal orientation to superimpose the RCC and the LCC, and display the NCC on the opposite side of the projection. This technique aims to reduce THV parallax and offer a more predictable ID. It is acknowledged that the occurrence of NOCD requiring PPI is directly linked to the MSL [16, 17]. Indeed, the conduction pathways protrude in the lower part of the membranous septum [30] and can be damaged by the deployment of the THV. Therefore, many observational studies on this subject demonstrated that COL may reduce PPI after TAVI, regardless of THV type [10, 11, 13, 15]. In a study limited to the SAPIEN 3 BEV (Edwards Lifescience), Sammour et al. [10] found that a systematic high deployment approach using the COL reduced both PPI (5.5% vs. 13.1%; *p* < 0.01) and NOP-LBBB (5.3% versus 12.2%; *p* < 0.01) at 30 days. Similarly, in a large cohort involving 995 propensity-matched pairs of patients who underwent TAVI with the self-expanding Evolut Platform (Medtronic), the rate of PPI at discharge was lower in COL group than in the conventional technique group (17.0 vs. 11.9%; *p* = 0.001), yet more NOP-LBBB were observed in the COL group (27.4% vs. 22.0%; *p* = 0.01), questioning the true independent effect of COL on the reduction of PPI from a pathophysiological standpoint. Most previous studies compared more contemporary patients treated with the COL technique to their “historical” TCC counterparts, which introduces significant biases that cannot be adequately adjusted for in statistical analyses. Indeed, increased operators’ awareness of the detrimental effect of post-TAVI PPI and the publication of experts’ consensus and international guidelines [21] during the inclusion period of several of these studies likely influenced both the performance of TAVI procedures and immediate post-procedural care regarding NOCD [11–13]. We did not demonstrate that using the COL technique per se leads to a significant reduction of HAVB or PPI at 30 days. Nevertheless, the COL technique was associated with a lower ID, resulting in less contact between the lower part of the membranous septum and the THV. Secondly, a lower ∆MSID-NCC significantly reduced PPI at 30 days. Therefore, COL may facilitate procedural conditions that favor higher implantation relative to the membranous septum. Furthermore, it should be highlighted that we were able to adequately adjust our analysis for major baseline (FD-AVB, RBBB), anatomical (bicuspid aortic valve, degree of calcification), and procedural (valve type, pre- and post-dilatation, year of the procedure) confounding factors to delineate the true independent effect of the COL technique [31, 32]. Moreover, although we also observed an increasing adoption of the COL technique, this was not associated with a trend toward higher implantation over time. Finally, the inclusion period of the present study was on the shorter side of those of previous studies and more importantly was subsequent to the publication of the first major expert consensus document guiding the management of post-TAVI NOCD [20].

The measurement of membranous septum length (MSL) in our study was performed using pre-procedural CT in a standardized coronal reconstruction, consistent with the methodology originally described by Hamdan et al. and subsequently adopted in multiple studies evaluating ΔMSID as a predictor of conduction disturbances [16]. In contrast to more recent approaches incorporating infra-annular reconstructions, percentage-based implantation metrics, or membranous septum area quantification [33–35], our method intentionally focused on a reproducible linear measurement aligned with the non-coronary cusp nadir, facilitating integration with angiographic implantation depth assessment. Similarly, implantation depth was measured on final angiography at the non-coronary cusp, in line with prior reports, including Jilaihawi et al.^34^, who emphasized the importance of implantation depth relative to the membranous septum in minimizing permanent pacemaker implantation after self-expanding TAVI. Although small absolute differences in implantation depth (≈ 1–2 mm) may appear modest, prior mechanistic studies suggest that such differences may be clinically meaningful given the close anatomical proximity of the conduction system to the virtual annular plane.

Sammour et al.^10^ reported an ID of 3.2 ± 1.9 mm using the TCC technique vs. 1.5 ± 1.6 mm with the COL technique in a cohort of BEV. ID in our cohort was closer to Pascual and Mendiz’s works, encompassing only SEV (5.14 ± 2.6 mm vs. 4.2 ± 2.1 mm and 5.65 ± 3.48 mm vs. 3.43 ± 2.79 mm, respectively) [11, 13]. Comparisons between studies remain difficult due to the different types of THV. Nonetheless, the small absolute differences in ID between the two techniques were comparable (approximately 1.6 mm). Nevertheless, these measurements remain subject to angiographic spatial resolution constraints (roughly 0.2 mm) and residual parallax, which should be considered when interpreting small between-group differences [9]. Interestingly, Sammour et al.^10^ reported a decreasing trend in ID in their population of TAVI recipients treated from April 2015 to December 2018. Of note, this trend was apparent before the implementation of the COL technique, and more pronounced in the TCC group (*p* < 0.001 vs. *p* = 0.052 in the COL group). This may reflect a paradigm shift that predates the use of COL, among operators, which aimed at a high implantation of THV to reduce NOCD and PPI.

In addition, very few studies reported the MSL, while it is an essential measurement in this context [16, 18, 36]. Firstly, in patients with short MSL, systematic high implantation could be insufficient to avoid NOCD, all the more so if other risk factors are associated [18, 28]. Secondly, high deployment might be useless or even detrimental to some patients with long MSL. Indeed, a recent study [37] demonstrated that a target ID between 1 and 3 mm reduced NOCD, but exposed to sinus sequestration in case of redo-TAVI and unfavorable future coronary access. These data suggest that the strategy of implantation should tend to a compromise between the risk of NOCD and the possibility of subsequent coronary access using a patient-specific multimodal approach to limit long-term adverse events. The most important limitation to the use of this measurement in daily practice is probably the absence of a standardized method and inter-observer variability [25].

The inclusion of multiple commercially available valve platforms reflects contemporary clinical practice and enhances the generalizability of our findings, as the primary objective of this study was to evaluate projection-based implantation strategies rather than device-specific performance. The observed association between Portico/Navitor implantation and conduction disturbances must be interpreted cautiously, as the patients included represent our initial institutional experience with this platform, corresponding to a limited number of procedures per operator. A learning-curve effect likely contributed to these findings. Importantly, larger contemporary series have reported permanent pacemaker implantation rates with the Navitor system comparable to other current-generation valves, suggesting that our findings should not be generalized beyond this early experience [38].

### Study limitations

This single-center retrospective study is subject to inherent limitations. First, operator-related selection bias in projection technique choice may play a significant role in our findings and cannot be adequately accounted for by statistical adjustment. Secondly, the measurements of the MSL and the ID were assessed retrospectively by a single expert operator thus not involving a central adjudication by a corelab. The intra-observer variability of ID measurement was not evaluated. Finally, given the modest absolute difference observed in the primary endpoint between groups, a risk of type II error cannot be ruled out. Indeed, a post-hoc power calculation demonstrated that more than 1300 patients would have been necessary in each group to demonstrate such a difference with an 80% power at the alpha level of 5%.

## Conclusion

The cusp-overlap technique does not independently reduce new-onset conduction disturbances after TAVI. However, it may facilitate higher implantation relative to the membranous septum length, which is a key anatomical determinant of conduction outcomes. These findings provide confirmatory evidence that implantation depth relative to membranous septum length remains a key anatomical determinant of conduction outcomes and underscore the importance of individualized, anatomy-guided implantation strategies rather than routine adoption of a specific projection technique.

### Clinical perspectives

- **What is known?** Conduction disturbances after TAVI are influenced by implantation depth and patient-specific anatomy.
- **What is New?** In a contemporary cohort, implantation depth relative to membranous septum length (ΔMSID) — rather than the angiographic projection strategy itself — was the key determinant of conduction outcomes. The cusp-overlap technique may serve as a facilitating imaging tool to achieve higher implantation but was not independently associated with long-term clinical outcomes.
- **What is Next?** Future studies should evaluate individualized, anatomy-guided implantation strategies balancing conduction risk, coronary access preservation, and redo-TAVI feasibility.

## Supplementary Information

Below is the link to the electronic supplementary material.

Supplementary Material 1

## Abbreviations

TAVI: Transcatheter Aortic Valve Implantation
NOCD: New-Onset Conduction Disturbances
PPI: Permanent Pacemaker Implantation
NOP-LBBB: New-Onset Persistent Left Bundle Branch Block
COL: Cusp-Overlap
TCC: Three Cusps Coplanar
LCC: Left Coronary Cusp
RCC: Right Coronary Cusp
NCC: Non Coronary Cusp
ID: Implantation Depth
MSL: Membranous Septum Length
BEV: Balloon Expendable Valve
SEV: Self Expandable Valve
