# Supplementary material for: Influence of The Cusp-Overlap and three cusps coplanar techniques on new-onset conduction disturbances following transcatheter aortic valve implantation
Source: Cardiovasc Interv Ther. 2026 Apr 4;41(3):770–82. doi: 10.1007/s12928-026-01276-0 (PMC13279278; doi:10.1007/s12928-026-01276-0)
Supplement: Supplementary file 1 — Supplementary Material 1 [file 12928_2026_1276_MOESM1_ESM.docx]

**Supplemental Appendix**

**Lemarchand L et al. Cusp-overlap technique in TAVI recipients**

**Tables**

**Table S1. Comparison of baseline characteristics between the primary outcome and no primary outcome groups.**

|  | **No primary outcome (n = 411)** | **Primary outcome (n = 90)** | **p-value** |
| --- | --- | --- | --- |
| Age, years | 79.9 (7.61) | 82.3 (5.94) | **<0.01** |
| Female, n | 195 (47%) | 38 (42%) | 0.37 |
| Weight, kg | 73.3 (16.1) | 72.2 (14.6) | 0.56 |
| Height, m | 1.64 (0.08) | 1.64 (0.08) | 0.89 |
| BMI, kg/m² | 27.1 (5.21) | 26.7 (4.53) | 0.53 |
| Euroscore II  Logistic, % | 10.6 (7.99) | 14.1 (12.6) | **0.02** |
| Hypertension, n | 307 (75%) | 68 (76%) | 0.86 |
| Diabetes mellitus, n | 77 (19%) | 21 (23%) | 0.32 |
| Previous CAD | 184 (45%) | 41 (46%) | 1 |
| Previous valvular intervention, n | 9 (2.2%) | 1 (1.1%) | 1 |
| Créatinine, µmol/L | 91.8 (53.7) | 103 (85.7) | 0.24 |
| Chronic Renal Disease, n | 113 (27%) | 31 (34%) | 0.19 |
| Chronic Pulmonary disease, n | 43 (10%) | 9 (10%) | 0.9 |
| ***Baseline ECG*** | | | |
| Atrial Fibrillation, n | 73 (18%) | 12 (13%) | 0.31 |
| PR interval (ms) | 182 (30.7) | 208 (56.5) | **<0.01** |
| FDAVB, n | 90 (27%) | 38 (49%) | **<0.01** |
| RBBB, n | 24 (5.8%) | 23 (26%) | **< 0.01** |
| LAHB, n | 60 (15%) | 19 (21%) | 0.12 |
| LBBB, n | 29 (7.1%) | 9 (10%) | 0.34 |
| ***MDCT data*** | | | |
| Membranous Septum Length, mm | 6.45 (2.07) | 5.74 (2.15) | **<0.01** |
| Annulus Area, mm² | 431 (86.3) | 448 (94.8) | 0.12 |
| Annulus Diameter, mm | 23.2 (2.33) | 23.6 (2.52) | 0.19 |
| Bicuspid Aortic Valve, n | 9 (2.2%) | 1 (1.1%) | 1 |
| Total volumes of calcifications, mm3 | 398 (325) | 478 (331) | **0.04** |
| % | 4.13 (3.32) | 4.89 (3.70) | 0.07 |
| DLZ, mm3 | 48.0 (54.7) | 61.9 (62.6) | 0.05 |
| % | 1.54 (1.64) | 1.93 (2.04) | 0.09 |
| DLZ-LCC | 14.2 (25.1) | 17.7 (26.8) | 0.26 |
| % | 1.43 (2.36) | 1.85 (2.96) | 0.21 |
| DLZ-NCC | 20.1 (29.5) | 26.4 (32.4) | 0.09 |
| % | 2.01 (2.81) | 2.38 (2.81) | 0.26 |
| DLZ-RCC | 13.7 (21.7) | 17.7 (31.3) | 0.26 |
| % | 1.21 (1.90) | 1.54 (2.79) | 0.28 |
| LVOT, mm3 | 24.8 (60.5) | 35.4 (70.5) | 0.19 |
| % | 0.398 (0.934) | 0.581 (1.30) | 0.21 |
| LVOT-LCC | 10.1 (36.5) | 13.6 (38.8) | 0.43 |
| % | 0.495 (1.55) | 0.786 (2.52) | 0.3 |
| LVOT-NCC | 11.4 (32.8) | 17.8 (38.2) | 0.14 |
| % | 0.571 (1.61) | 0.844 (1.98) | 0.22 |
| LVOT-RCC | 3.31 (11.6) | 4.04 (16.2) | 0.69 |
| % | 0.149 (0.563) | 0.186 (0.753) | 0.66 |
| Total Leaflet, mm3 | 373 (298) | 442 (303) | 0.05 |
| % | 4.89 (3.48) | 5.54 (3.40) | 0.11 |
| TL-LCC | 98.8 (103) | 118 (101) | 0.11 |
| % | 4.08 (3.78) | 4.71 (3.76) | 0.15 |
| TL-NCC | 159 (133) | 191 (140) | **< 0.05** |
| % | 6.43 (4.72) | 7.25 (4.61) | 0.13 |
| TL-RCC | 116 (112) | 133 (121) | 0.2 |
| % | 4.17 (3.76) | 4.67 (4.14) | 0.29 |
| Upper Leaflet, mm3 | 333 (272) | 393 (280) | 0.07 |
| % | 5.81 (4.26) | 6.54 (4.12) | 0.14 |
| UL-LCC | 87.8 (95.0) | 104 (93.0) | 0.13 |
| % | 4.81 (4.70) | 5.51 (4.49) | 0.19 |
| UL-NCC | 142 (122) | 170 (132) | 0.06 |
| % | 7.65 (5.83) | 8.63 (5.91) | 0.16 |
| UL-RCC | 104 (103) | 118 (111) | 0.26 |
| % | 4.97 (4.64) | 5.49 (4.99) | 0.36 |
| ***Echocardiographic Parameters*** | | | |
| Mean Gradient, mmHg | 55.5 (17.7) | 57.5 (14.5) | 0.26 |
| Vmax, m/s | 4.57 (0.710) | 4.70 (0.618) | 0.08 |
| Indexed Surface, cm²/m² | 0.411 (0.119) | 0.399 (0.0854) | 0.28 |
| LVEF, % | 62.0 (11.4) | 61.1 (11.5) | 0.51 |
| Indexed LVTDD, mm/m² | 25.7 (4.23) | 26.1 (4.48) | 0.42 |
| Low Flow/Low Gradient, n | 32 (7.9%) | 2 (2.2%) | 0.05 |

BMI, Body Mass Index; CAD, Coronary Artery Disease; DLZ, Device Landing Zone; ECG, Electrocardiogram; FD-AVB, First Degree Atrio-Ventricular Block; LAHB, Left Anterior Hemi-Block; LBBB, Left Bundle Branch Block; LCC, Left Coronary Cusp; LVEF, Left Ventricular Ejection Fraction; LVTDD, Left Ventricular Telediastolic Diameter; LVOT, Left Ventricular Outflow Tract; MDCT, Multidetector Computed Tomography; mmHg, millimeters of Mercury; NCC, Non Coronary Cusp; PPI, Permanent Pacemaker Implantation; RCC, Right Coronary Cusp; UL, Upper Leaflet; Vmax, peak velocity.

**Table S2. Comparison of the procedural and post-procedural data of the primary outcome and the no primary outcome groups.**

|  | **No Primary Outcome (n = 411)** | **Primary Outcome (n = 90)** | **p-value** |
| --- | --- | --- | --- |
| Femoral Access, n | 365 (89%) | 82 (91%) | 0.52 |
| Carotid Access, n | 46 (11%) | 8 (8.9%) | 0.52 |
| Predilatation, n | 211 (51%) | 51 (57%) | 0.36 |
| Post-dilatation, n | 36 (8.8%) | 7 (7.8%) | 0.76 |
| Cusp-Overlap, n | 150 (36%) | 27 (30%) | 0.24 |
| Three-Cusp Coplanar, n | 261 (64%) | 63 (70%) | 0.24 |
| ***THV*** | | | |
| BEV, n | 316 (77%) | 63 (70%) | 0.17 |
| SEV, n | 94 (23%) | 27 (30%) | 0.17 |
| Edwards, n | 316 (77%) | 63 (70%) | 0.17 |
| CoreValve, n | 55 (13%) | 12 (13%) | 0.99 |
| Portico, n | 10 (2.4%) | 3 (3.3%) | 0.71 |
| Acurate NEO, n | 14 (3.4%) | 1 (1.1%) | 0.49 |
| Navitor, n | 15 (3.6%) | 11 (12%) | **< 0.01** |
| ***THV Size*** |  |  |  |
| 20, n | 8 (1.9%) | 0 (0%) | 0.36 |
| 23, n | 134 (33%) | 19 (21%) | **0.03** |
| 25, n | 18 (4.4%) | 2 (2.2%) | 0.55 |
| 26, n | 153 (37%) | 30 (33%) | 0.49 |
| 27, n | 8 (1.9%) | 7 (7.8%) | **< 0.01** |
| 29, n | 83 (20%) | 31 (34%) | **< 0.01** |
| 34, n | 7 (1.7%) | 1 (1.1%) | 1 |
| Area oversizing, % | 13.8 (1.8-27.5) | 9.5 (0.1-25.6) | 0.13 |
| ***Depth Implantation*** | | | |
| LCC-THV, mm | 4.60 (2.15) | 5.95 (2.57) | **<0.01** |
| Mean NCC – LCC, mm | 4.62 (1.99) | 6.26 (2.31) | **<0.01** |
| NCC-THV, mm | 4.66 (2.33) | 6.56 (2.66) | **<0.01** |
| ∆MSID-LCC, mm | 1.85 (2.98) | -0.315 (3.49) | **<0.01** |
| ∆MSID mean LCC – NCC, mm | 1.82 (2.85) | -0.522 (3.36) | **<0.01** |
| ∆MSID-NCC, mm | 1.79 (3.08) | -0.819 (3.57) | **<0.01** |
| ***Echocardiographic Parameters*** | | | |
| Mean Gradient, mmHg | 12.0 (5.16) | 11.2 (5.26) | 0.22 |
| Vmax, m/s | 2.24 (0.474) | 2.19 (0.452) | 0.33 |
| Permeability Index, % | 54.0 (14.5) | 52.6 (14.7) | 0.46 |
| LEVF, % | 62.7 (9.75) | 61.8 (9.82) | 0.47 |
| Indexed Surface, mm/m² | 1.18 (0.419) | 1.17 (0.427) | 0.79 |
| ***Follow-up*** | | | |
| Follow-up, months | 18.0 (11.9) | 15.7 (12.3) | 0.12 |
| Rehospitalization for HF, n | 34 (9.8%) | 9 (11%) | 0.72 |
| Death, n | 21 (5.8%) | 14 (17%) | **<0.01** |

**Figures**

**Figure S1. Bland-Altman analysis of the intra-observer variability of the membranous septum length measurement.**

**
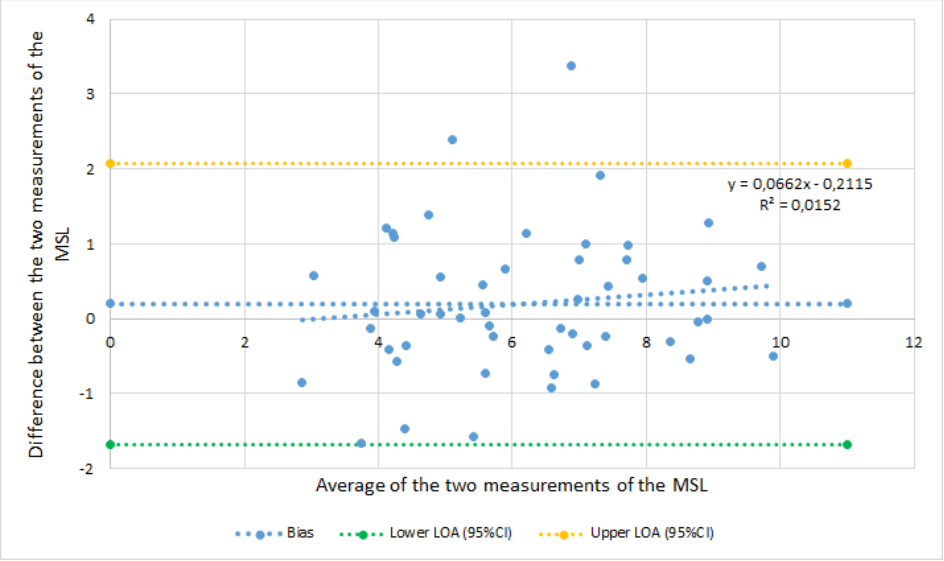
**

CI, confidence interval; LOA, limits of agreement; MSL, membranous septum length. Bland-Altman analysis showed a bias of 0.2 mm (95% limits of agreement (LOA): -1.68 to 2.08) between the two successive measurements. Lin’s concordance coefficient was 0.86 (95% LOA: 0.77; 0.92), corresponding to an “almost perfect” correlation.

**Figure S2. Segmentation of the aortic valve complex**

Upper leaflet (a), Left Ventricular Outflow Tract (b), and the Device Landing Zone (c). Each section was subdivided into 3 sectors according to the 3 coronary cusps (d).


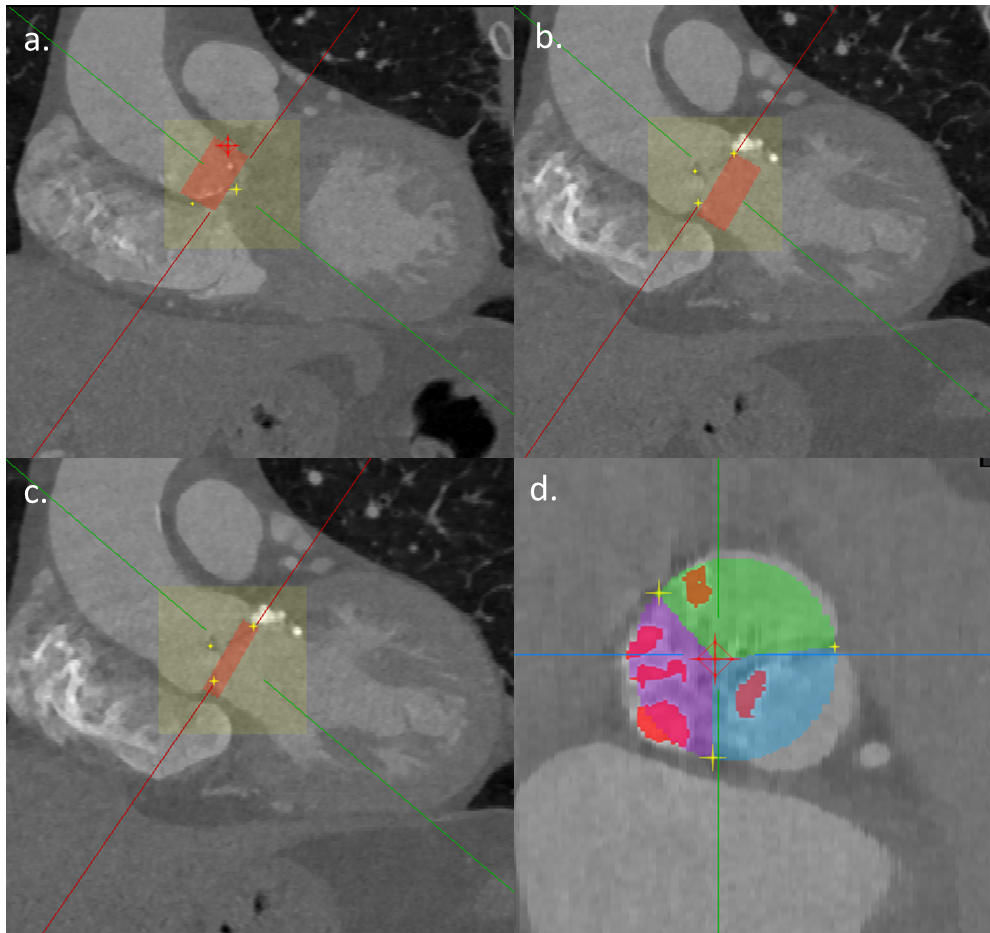


**Figure S3. Flow Chart of the study population**

**
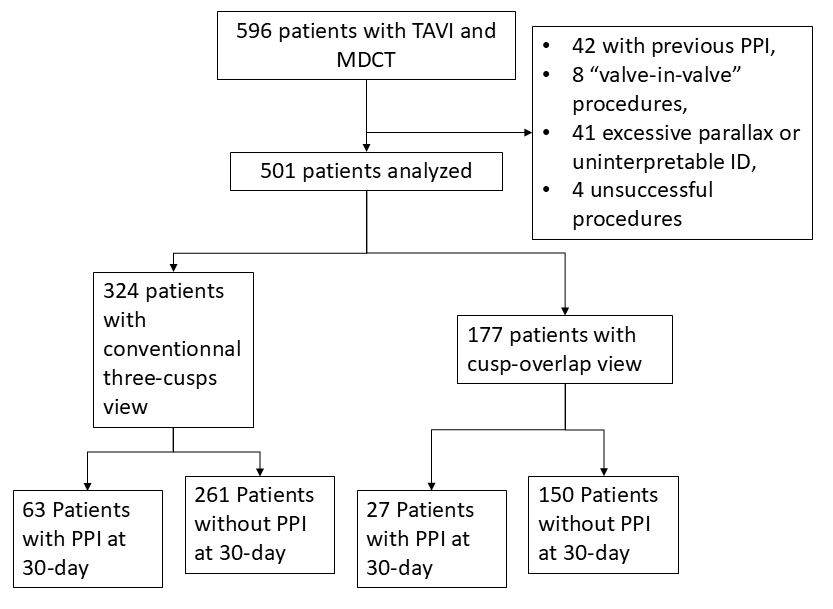
**

MDCT, Multidetector Computed Tomography; ID, Implantation Depth; PPI, Permanent Pacemaker Implantation; TAVI, Transcatheter Aortic Valve Implantation.

**Figure S4. Repartition of the Membranous Septum Length in the overall population.**


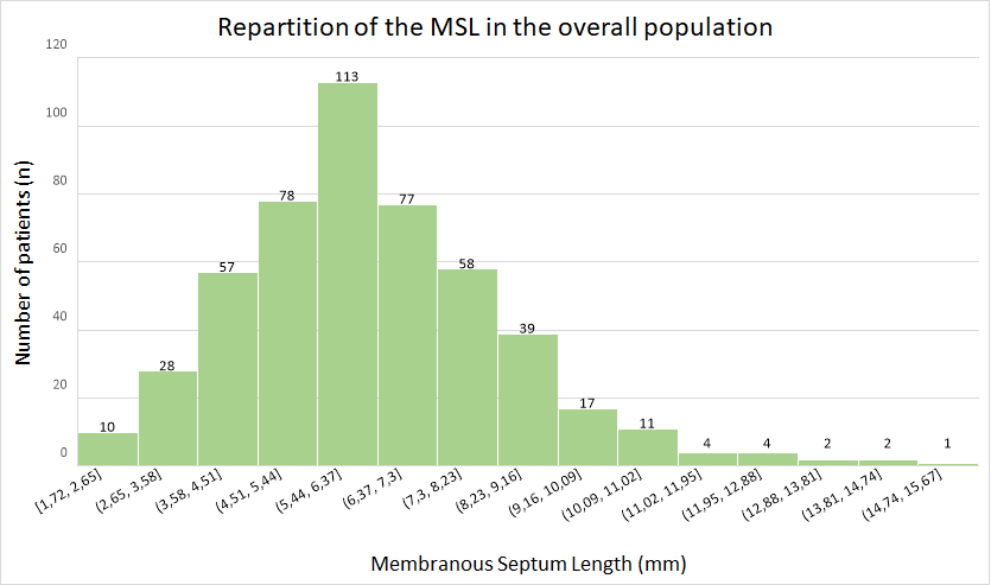


**Figure S5. Relationship between the implantation depth of the transcatheter heart valve and the occurrence of the primary outcome and the technique of implantation.**

**
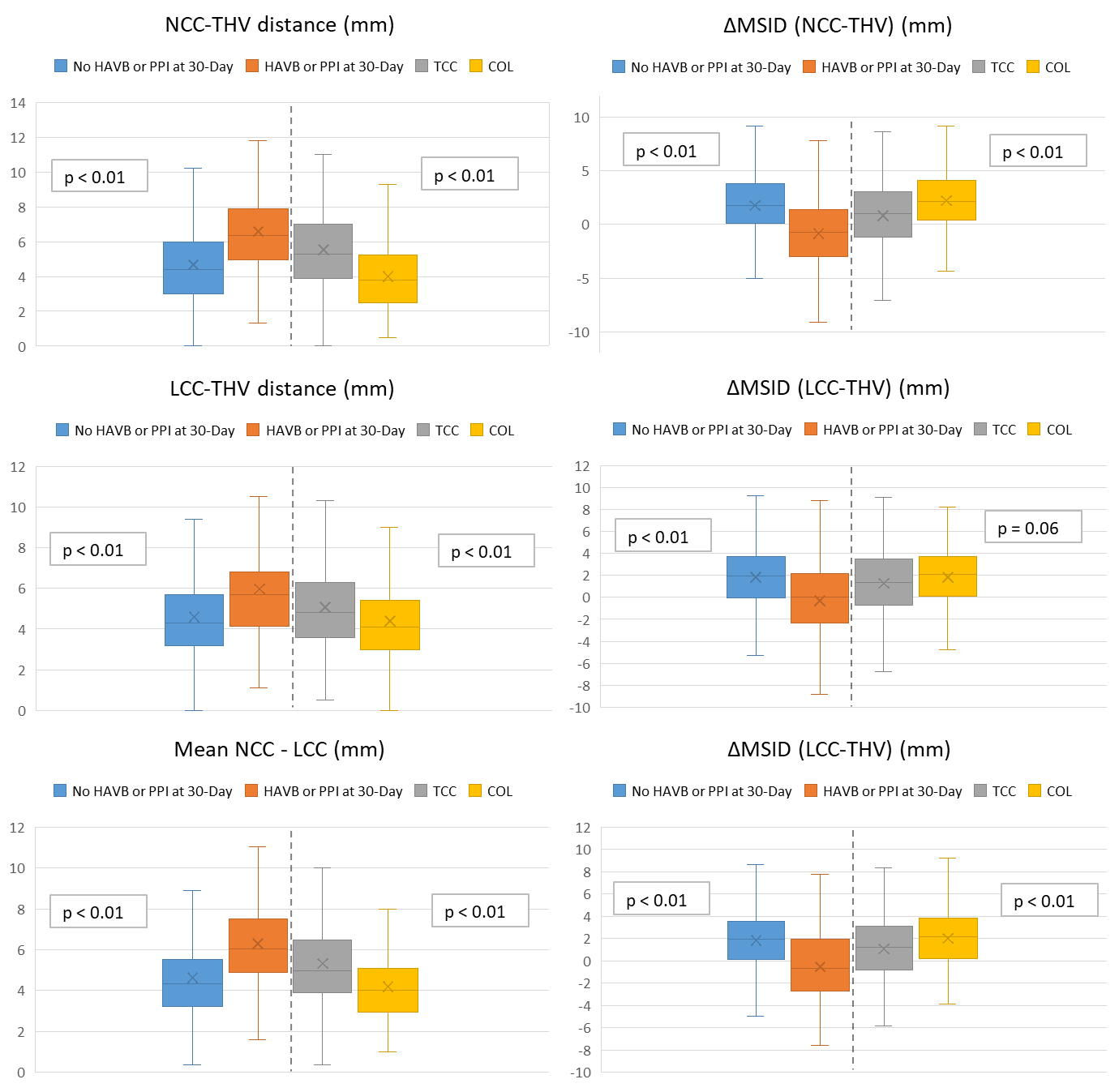
**

COL, Cusp Overlap; ∆MSID, delta between Membranous Septum length and Implantation Depth; HAVB, High-degree Atrioventricular Block; LCC, Left Coronary Cusp; NCC, Non-Coronary Cusp; PPI, Permanent Pacemaker Implantation; RCC, Right Coronary Cusp; TCC, Three Cusps Coplanar; THV, Transcatheter Heart Valve.

**Figure S6. Standardized differences for each baseline variable before (blue spots) and after propensity score overlap weighting (red triangles).**


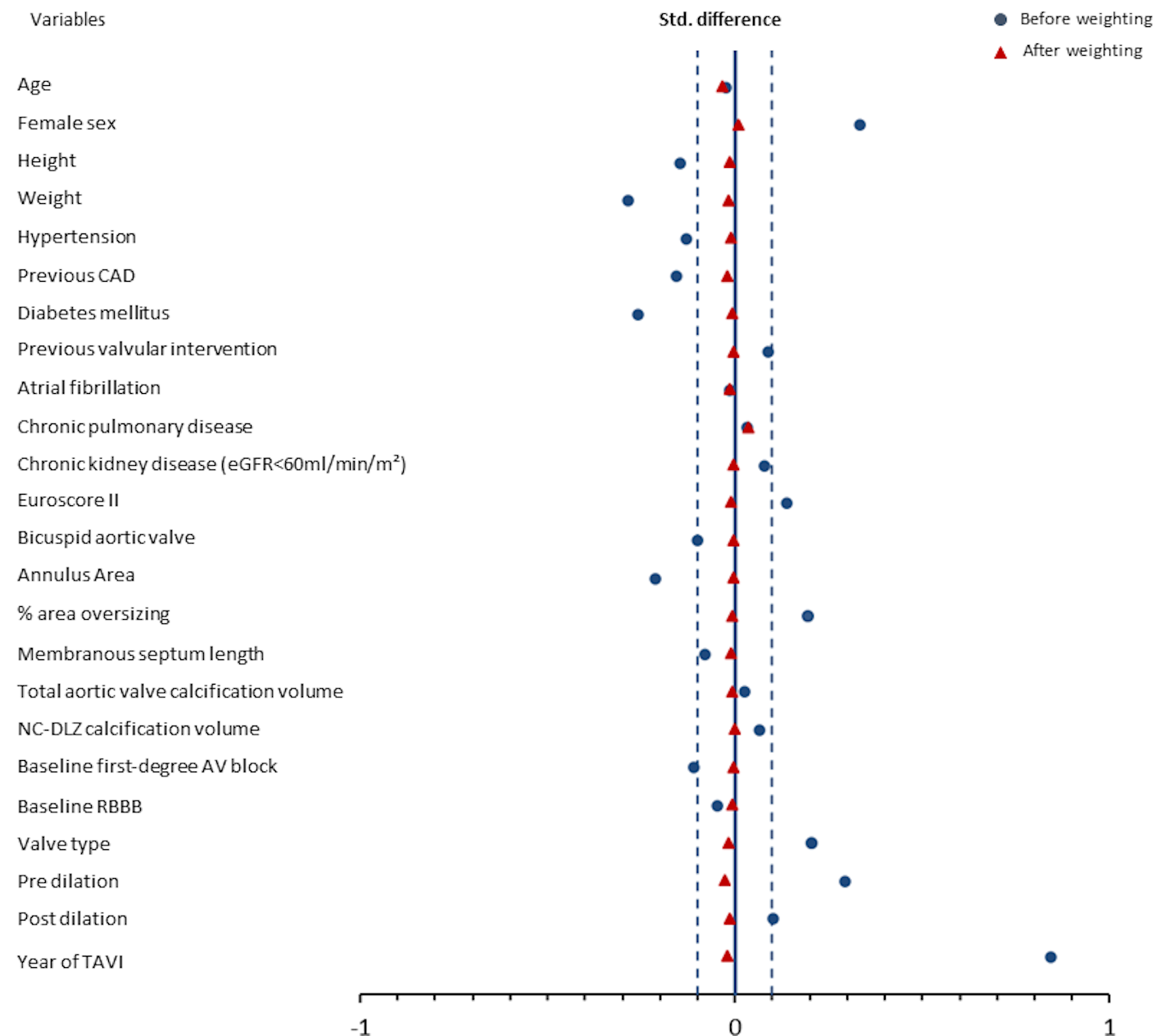


Variables with standardized differences between -0.10 and 0.10 are considered well-balanced (blue dotted lines). AVB; Atrio-Ventricular Block; CAD, Coronary Artery Disease; DLZ, Device Landing Zone; eGFR, estimated Glomerular Flow Rate; NCC, Non Coronary Cusp; RBBB, Right Bundle Branch Block; TAVI, Transcatheter Aortic Valve Implantation.
